# New Publications

**Published:** 1900-12

**Authors:** 


### NEW PUBLICATIONS.

THE COLUMBIA CALENDAR.

The Columbia Calendar, issued by the Pope Manufacturing Company, of Hartford, Conn., has once again reached our desk for the year ushering in the new century. Always bright and sparkling with fresh thoughts and expressions from those best acquainted with the merits of their products, it fills a doubly useful place on one's desk as a daily reminder of one's engagements, etc.

PHYSICIANS' VISITING LIST.

It rarely falls to the lot of any one type of book to be published for so long a period as fifty years, yet this is just what has been the experience of the *Physicians' Visiting List*, published by Messrs. P. Blakiston's Son & Co., of Philadelphia. It is not to-day just like the one published fifty years ago, but it has grown in value and merit with each year, until those who have grown familiar with its usefulness would no more think of starting a new year without its assistance than they would go without a well-equipped medicine-chest for emergencies.

